# Combining waterlase ablation and submucosal ALA injection to enhance photodynamic therapy in early-stage oral squamous cell carcinoma: a three-patient case series

**DOI:** 10.1186/s12903-026-07731-x

**Published:** 2026-01-26

**Authors:** Fumin Zheng, Lu Dong, Lin Han, Yaozhong Wang, Xixi Yu, Wanchun Wang

**Affiliations:** 1 Department of Stomatology, Qingdao Stomatological Hospital Affiliated to Qingdao University, No. 17 Dexian Road, Qingdao, 266005 China; 2 School of Stomatology, Qingdao University, No. 380 Ningxia Road, Qingdao, 266000 China; 3 Department of Stomatology, Qingdao Women and Children’s Hospital Affiliated to Qingdao University, No.6 Tongfu Road, Qingdao, 266000 China; 4 Department of Oral Mucosal disease, Qingdao Stomatological Hospital Affiliated to Qingdao University, No. 17 Dexian Road, 266005 Qingdao, China

**Keywords:** Photodynamic therapy, Oral squamous cell carcinoma, Waterlase, ALA, DNA-ICM

## Abstract

Oral squamous cell carcinoma (OSCC), a common type of oral cancer, has traditionally relied on surgery or radiotherapy as the main treatment. However, these treatments often put patients at risk of deformity and speech dysfunction due to the special anatomical location of the tongue. Local 5-aminolevulinic acid photodynamic therapy (ALA-PDT) is an effective and non-invasive in situ treatment method that can treat tumors while preserving normal tissue morphology and structure. Nevertheless, the curative effect of ALA-PDT is constrained by the permeability of the photosensitizer and the penetration depth of the therapeutic laser source. This study reports three cases of early-stage well-differentiated OSCC that were managed with a nonsurgical approach. In an attempt to enhance the therapeutic effectiveness of ALA-PDT, we employed Waterlase technology to perform abrasion on the lesioned area, with the dual goals of eliminating local hyperplasia and white lesions and facilitating deeper penetration of the laser light source. Immediately following this, these patients underwent local puncture-like injections of 20% ALA. By means of this procedure, the interference caused by the epithelium on the penetration of ALA could be directly avoided. Upon completion of a 2 h incubation period, PDT was initiated at a dose of 119.4 J/cm². Before and after treatment, we evaluated the treatment effect by artificial intelligence (AI)-assisted deoxyribo nucleic acid (DNA) aneuploidy cytology by image cytometry (DNA-ICM). After 6 months of follow-up observation, these patients showed no signs of tumor recurrence. This preliminary case series suggests that Waterlase ablation-assisted submucosal ALA-PDT may provide a potential treatment alternative for early-stage OSCC. Notably, it particularly shines in the aspects of maintaining the patient’s normal physiological functions and elevating the quality of life.

All patients provided linguistically appropriate written informed consent after receiving detailed information about ALA-PDT’s therapeutic mechanisms, recurrence risks, and alternative therapies.

## Introduction

Oral squamous cell carcinoma (OSCC) accounts for over 90% of malignant oral neoplasms and represents a significant clinical challenge due to its aggressive biology and high mortality rate among head and neck cancers [1]. While surgical resection and radiotherapy remain standard curative modalities for early-stage disease, their application in tongue-specific OSCC carries substantial functional morbidity—including impaired speech, dysphagia, restricted tongue mobility, and aesthetic compromise—prompting exploration of tissue-preserving alternatives [2, 3].

Recent advances in precision oncology have positioned photodynamic therapy (PDT) as a promising non-invasive strategy, particularly 5-aminolevulinic acid (ALA)-PDT, which exploits tumor-selective accumulation of protoporphyrin IX to induce targeted phototoxicity through subcellular membrane disruption and oxidative damage [4]. ALA administration for PDT can be achieved through systemic (oral/IV) or intratumoral routes. Systemic delivery provides broad distribution but causes prolonged photosensitivity and variable tumor accumulation due to poor penetration. In contrast, intratumoral ALA injection delivers higher local concentrations directly to the tumor site, minimizing systemic exposure and photosensitivity while enhancing therapeutic precision. For early OSCC, intratumoral administration is particularly advantageous due to the anatomical accessibility of oral lesions and the need for targeted treatment with reduced side effects. Clinical evidence supports its efficacy for oral premalignant lesions and carcinoma in situ [5]; however, suboptimal ALA penetration depth and photosensitizer bioavailability in dense epithelial tissues have limited its utility for invasive OSCC [6].

To overcome these barriers, we explored an integrated approach combining Waterlase ablation-assisted submucosal ablation (Waterlase^®^, Biolase) with ALA-PDT—a technique aimed at addressing both the physical barrier of the lesion and the pharmacokinetic challenges of ALA delivery. Herein, we present three-patient case series successfully managed with this novel protocol who declined conventional surgery, demonstrating complete remission with sustained genomic stability via DNA image cytometry (DNA-ICM) monitoring.

## Materials and methods

### Patient selection criteria

Patients were selected based on the following inclusion criteria: histologically confirmed early OSCC staged as cT1-2N0M0 according to the 8th edition of AJCC/UICC TNM staging system, with tumor infiltration depth ≤ 2 mm on preoperative biopsy assessment. Exclusion criteria included: lesions involving bone or major neurovascular structures, history of prior radiotherapy or chemotherapy to the head and neck region, pregnancy or lactation, known hypersensitivity to ALA or its derivatives, and systemic conditions contraindicating laser treatment.

### Equipment and technical parameters

Waterlase ablation was performed using an Er, Cr: YSGG laser system (Biolase Technology Inc., Irvine, CA) operating at a wavelength of 2780 nm. The laser was calibrated to deliver 1.5 W power with a pulse duration of 140 µs at 35 Hz frequency, using a non-contact technique with continuous fiber tip movement for precise epithelial tissue removal. For ALA-PDT, a diode laser system (Wuhan Yage Optic and Electronic Technique Co., Ltd, China) with wavelength of 635 nm, output power of 375 mW, spot diameter of 2 cm (power density: 119.4 mW/cm²), and irradiation time of 1000 s (energy density: 119.4 J/cm²) was employed. Fluorescence guidance was provided by VELscope Vx Handpiece (LED Dental Inc., Canada).

### ALA administration protocol

**Scheme 1:**
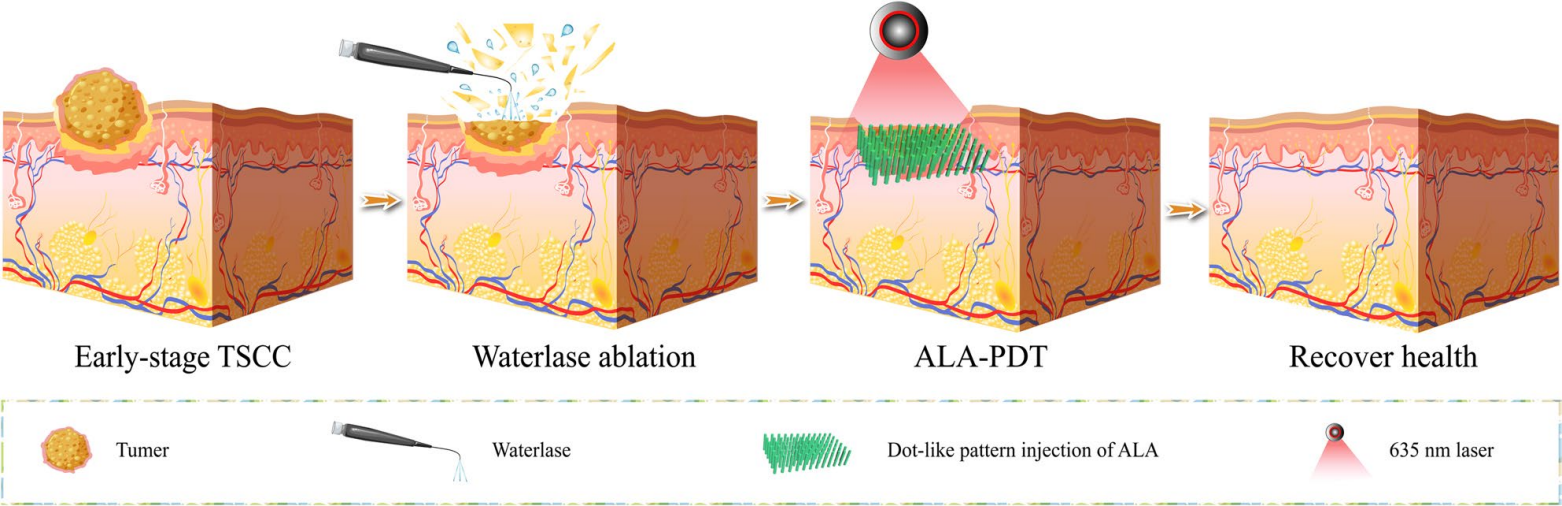
Schematic illustration of Waterlase ablation-assisted submucosal ALA-PDT for early-stage OSCC

A 20% ALA solution was prepared by dissolving 118 mg of 5-aminolevulinic acid hydrochloride (ALA-HCl) powder (Shanghai Fudan Zhangjiang Biopharmaceutical Co., Ltd.) in 0.59 ml of 0.9% normal saline. Immediately following Waterlase ablation, the ALA solution was administered via submucosal injection in a dot-like pattern using a standard 1 ml syringe with conventional hypodermic needle, with injection points spaced approximately 1 mm apart across the entire lesion area plus 2–3 mm margins (Scheme 1). The solution was retained for 2 h to allow optimal protoporphyrin IX accumulation before laser irradiation.

### Evaluation methods

Histopathological examination was conducted on pre-treatment incisional biopsies and post-treatment specimens using hematoxylin-eosin staining to assess tumor differentiation, infiltration depth, and pathological response. DNA image cytometry (DNA-ICM) was performed using AI-assisted image analysis (Wuhan Landing Cloud Clinical Laboratory Co., Ltd.) to quantify aneuploid cells (DNA index ≥ 2.5c) as biomarkers of malignant transformation. Imaging evaluation included contrast-enhanced ultrasonography and CT scanning to assess local invasion and rule out regional metastasis.

### Outcome measures

Primary outcomes: The clinical complete response rate and pathological complete response rate following single-session Waterlase ablation combined with submucosal 5-ALA-PDT treatment. These will be assessed through: (1) post-treatment visual clinical examination, and (2) DNA image cytometry (DNA-ICM) quantifying the reduction in aneuploid cells (DNA index ≥ 2.5c) as an objective biomarker of tumor cell clearance.

Secondary outcomes: (1) Safety and tolerability profiles, defined as the absence of significant bleeding, infection, or functional impairment (particularly speech and swallowing dysfunction); (2) Recurrence-free survival rates at 1-month, 3-month and 6-month follow-up intervals, evaluated through combined DNA-ICM analysis and clinical examination; and (3) Patient-reported outcomes including tongue function preservation and patient-reported outcomes including pain scores (VAS).

## Case 1

**Fig. 1:**
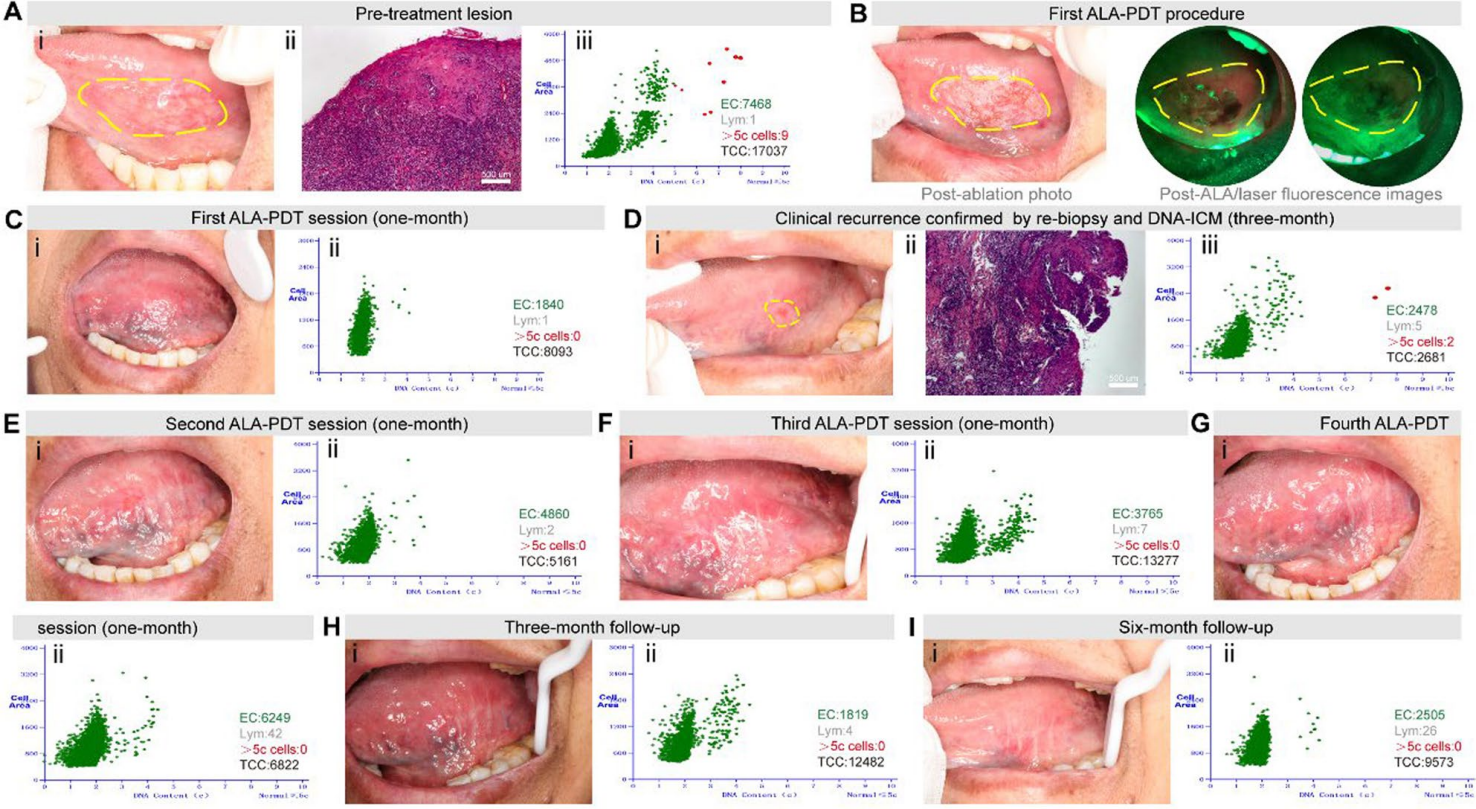
Clinical and pathological findings of Patient 1. **A**. (i) Patient 1’s lingual photo before treatment showing ulcerative lesion, (ii) Histopathology (100×) of initial biopsy revealing early-stage Oral Squamous Cell Carcinoma (OSCC); (iii) Artificial intelligence (AI)-assisted DNA aneuploidy cytology by image cytometry (DNA-ICM) analysis confirming aneuploidy; **B**. Post-ablation photograph and fluorescence imaging after ALA application and laser irradiation; **C**. (i) Lingual photograph at 1-month follow-up after initial 5-aminolevulinic acid photodynamic therapy (ALA-PDT) session, demonstrating complete ulcer re-epithelialization; (ii) DNA-ICM analysis demonstrated restoration of diploid DNA content; **D**. (i) Lingual photograph at 3-month follow-up demonstrating clinical recurrence; (ii) Re-biopsy histopathology (100×) confirming recurrent lesion; (iii) DNA-ICM revealed significant aneuploidy, indicating genomic instability and high-risk malignant transformation; **E**. (i) Photo of lingual taken at 1-month after second ALA-PDT session; (ii) Corresponding DNA-ICM analysis; **F**. (i) Photo of lingual taken at 1-month after third ALA-PDT session; (ii) Corresponding DNA-ICM analysis; **G**. (i) Photo of lingual taken at 1-month after fourth ALA-PDT session; (ii) Corresponding DNA-ICM analysis; **H**. (i) Photo of lingual taken at 3-month (post-fourth session): no recurrence, smooth mucosal surface; (ii) DNA-ICM quantified diploid DNA content, consistent with absence of malignant transformation and therapeutic efficacy; **I**. i. Photo of lingual taken at 6-month (post-fourth session): complete re-epithelialization, homogeneous pink mucosa, smooth texture without keratosis, and absent spontaneous bleeding; ii. Serial DNA-ICM demonstrated persistent genomic stability. EC: Epithelial cells; Lym: Lymphocytes;>5c cells: DNA content (c) = 5 (5c) is generally regarded as the boundary of diseased cells - that is, cells with >5c are diseased cells (marked in red circles on the chart); TCC: Total cell counts

A 72-year-old female presented a white streak, erosion and non-nodular region on the left lingual margin, progressively developing over a period exceeding one year. The lesion featured a soft texture and clear boundaries, showing an irregular oval shape of 35 mm × 18 mm in size (Fig. 1A i), with baseline pain rated 5/10 on the VAS. Histopathologic analysis of tissue samples taken from the most representative areas of the lesion (either central or peripheral regions) verified a highly differentiated squamous cell carcinoma at an early stage, with an infiltration depth of approximately 1 mm 0.4 ± 0.1 mm, mean ± standard deviation) (Fig. 1A ii). Artificial intelligence (AI)-assisted DNA aneuploidy cytology by image cytometry revealed the presence of multiple cells with abnormal DNA aneuploid (Fig. 1A iii) [7]. CT and ultrasound confirmed the absence of local metastasis. Given that the patient was elderly and harbored concerns that tongue resection might impact pronunciation and functions, she resolutely declined direct surgical resection. As a result, the patient chose non-surgical treatment, and in light of the clinical manifestations, we performed Waterlase ablation-assisted submucosal ALA-PDT. Firstly, the mucosal hyperplasia and white lesions were thoroughly removed by means of Waterlase under local anesthesia (Fig. 1B). In ALA-PDT, a 20% ALA solution, was injected in a dot-like pattern with a spacing of approximately 1 mm and retained for 2 h. For the three early OSCC cases in this study, ALA was injected at a standardized depth of 2–3 mm, extending 1–2 mm beyond the maximum tumor thickness to ensure complete lesion coverage with microscopic margins. Thanks to the VELscope Vx Handpiece, the presence of fluorescence staining within the lesion area could be identified. Subsequently, the lesion was irradiated with a laser. Upon the completion of the treatment, an assessment was carried out using the VELscope, and it was detected that the fluorescence had disappeared (Fig. 1B). Notably, for lesions exceeding the laser spot diameter, we employed a systematic overlapping irradiation technique guided by real-time VELscope fluorescence imaging to monitor photobleaching at margins and adjusted irradiation parameters until complete fluorescence elimination was achieved across the entire lesion area. Workflow schematic for treatment according to Scheme 1. After the ALA-PDT, extensive erosion appeared on the ventral surface of the patient’s tongue within two weeks, with peak VAS pain of 8/10 at day 4, gradually decreasing to 4/10 by day 14. After one session, the ulcer showed complete re-epithelialization at 1-month follow-up (Fig. 1C. i), with VAS pain reduced to 0/10. DNA-ICM showed restoration of diploid DNA content (Fig. 1C. ii), indicating a positive initial response. At 3-month, clinical recurrence was observed (Fig. 1D. i), with VAS pain increasing to 4/10. Re-biopsy (Fig. 1D. ii) confirmed recurrent OSCC, and DNA-ICM demonstrated significant aneuploidy (Fig. 1D. iii), indicating malignant transformation. Three additional ALA-PDT sessions were administered, with each session showing similar pain patterns. At 1-month post-second (Fig. 1E), VAS was 2/10; post-third (Fig. 1F), VAS was 1/10; and post-fourth (Fig. 1G) sessions, VAS was 0/10, with DNA-ICM showing progressive diploid restoration. At 3 months post-fourth session (Fig. 1H), the mucosa was smooth with no recurrence, and DNA-ICM confirmed diploid DNA content. At 6 months (Fig. 1I), the lingual surface exhibited complete re-epithelialization, homogeneous pink mucosa, and persistent genomic stability (DNA-ICM), with sustained pain-free status (VAS 0/10).

## Case 2

**Fig. 2:**
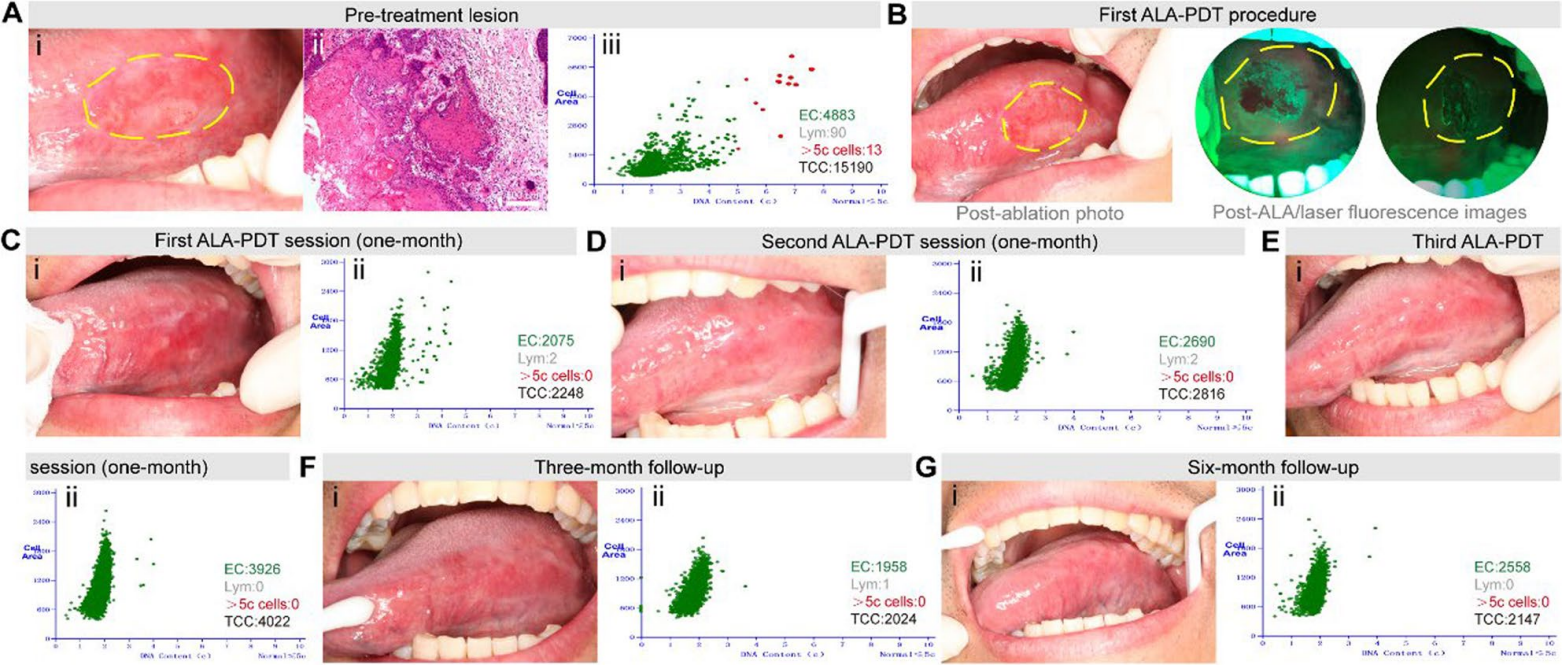
Clinical and pathological findings of Patient 2. **A** i. Patient 2’s lingual photo before treatment showing ulcerative lesion, ii. Histopathology (100×) of initial biopsy revealing early-stage OSCC; iii. DNA-ICM analysis confirming aneuploidy; (**B**) Post-ablation photograph and fluorescence imaging after ALA application and laser irradiation; (**C**) (i) Lingual photograph at 1-month follow-up after first ALA-PDT session, demonstrating partial re-epithelialization (80%); (ii) DNA-ICM analysis demonstrated restoration of diploid DNA content; **D**. (i) Photo of lingual taken at 1-month after second ALA-PDT session; (ii) Corresponding DNA-ICM analysis; **E**. (i) Photo of lingual taken at 1-month after third ALA-PDT session; (ii) Corresponding DNA-ICM analysis; **F**. (i) Photo of lingual taken at 3-month (post-third session): no recurrence, smooth mucosal surface; (ii) DNA-ICM quantified diploid DNA content, consistent with absence of malignant transformation and therapeutic efficacy; **G**. (i) Photo of lingual taken at 6-month (post-third session): complete re-epithelialization, homogeneous pink mucosa, smooth texture without keratosis, and absent spontaneous bleeding; (ii) Serial DNA-ICM demonstrated persistent genomic stability

A 34-year-old male presented with a persistent lingual ulcer measuring 22 mm × 12 mm (Fig. 2A. i), reporting baseline pain of 7/10 on the VAS, confirmed as early-stage OSCC via biopsy showing histopathological infiltration depth of 0.7 mm (0.3 ± 0.1 mm) (Fig. 2A. ii), with DNA-ICM revealing pathologic aneuploidy (> 5c cells) (Fig. 2A. iii). During the initial ALA-PDT session, hyperplastic mucosal tissue was ablated with Waterlase, and subsequent VELscope evaluation verified the elimination of fluorescence (Fig. 2B). After initial ALA-PDT, partial re-epithelialization (80%) and transient diploid restoration were observed at 1 month (Fig. 2C), with VAS pain score of 2/10, prompting two additional sequential sessions per the multi-session protocol informed by prior recurrence risks. Complete clinical resolution and persistent diploid stability emerged progressively through the second (Fig. 2D; VAS 0/10) and third sessions (Fig. 2E; VAS 0/10), culminating in a smooth mucosal surface at 3 months (Fig. 2F. i) with diploid DNA-ICM (Fig. 2F. ii) and VAS 0/10, and at 6 months (Fig. 2G), homogeneous pink mucosa with absent keratosis and sustained genomic stability with no pain (VAS 0/10)—demonstrating that three ALA-PDT sessions achieved durable remission without recurrence, validating the prophylactic multi-session approach to eradicate latent malignant clones despite early biomarker normalization.

## Case 3

**Fig. 3:**
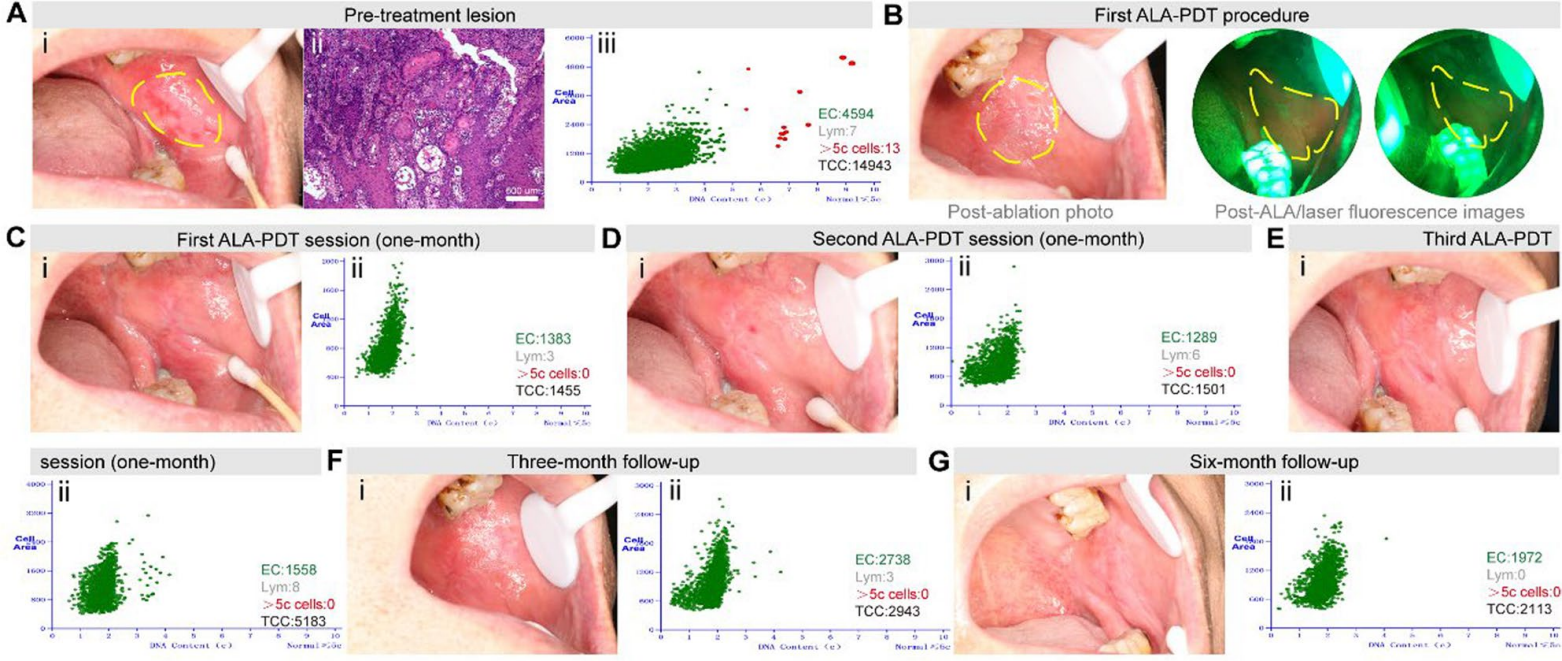
Clinical and pathological findings of Patient 3. **A** i. Patient 3’s buccal mucosa photo showed ulceration lesion before treatment; ii. Histopathology (100×) of initial biopsy revealing early-stage OSCC; iii. DNA-ICM analysis confirming aneuploidy; (**B**) Post-ablation photograph and fluorescence imaging after ALA application and laser irradiation; (**C**) (i) Buccal mucosa photograph at 1-month follow-up after first ALA-PDT session, demonstrating partial re-epithelialization (95%); (ii) DNA-ICM analysis demonstrated restoration of diploid DNA content; **D**. (i) Photo of buccal mucosa taken at 1-month after second ALA-PDT session; (ii) Corresponding DNA-ICM analysis; **E**. (i) Photo of buccal mucosa taken at 1-month after third ALA-PDT session; (ii) Corresponding DNA-ICM analysis; **F**. (i) Photo of buccal mucosa taken at 3-month (post-third session): no recurrence, smooth mucosal surface; (ii) DNA-ICM quantified diploid DNA content, consistent with absence of malignant transformation and therapeutic efficacy; **G**. (i) Photo of buccal mucosa taken at 6-month (post-third session): complete re-epithelialization, homogeneous pink mucosa, smooth texture without keratosis, and absent spontaneous bleeding; (ii) Serial DNA-ICM demonstrated persistent genomic stability

A 69-year-old male presented with a persistent buccal mucosa ulcer measuring 25 mm × 15 mm with baseline pain VAS 8/10 (Fig. 3A. i), confirmed as early-stage OSCC via biopsy showing histopathological infiltration depth of 0.9 mm (0.3 ± 0.1 mm) (Fig. 3A. ii), with DNA-ICM revealing pathologic aneuploidy (> 5c cells) (Fig. 3A. iii). Under local anesthesia, mucosal hyperplasia was removed using Waterlase, followed by VELscope assessment confirming the disappearance of fluorescence (Fig. 3B). After initial ALA-PDT, pain reduced to VAS 3/10 at 1-month follow-up with partial re-epithelialization (95%) and transient diploid restoration (Fig. 3C); however, recurrent pain (VAS 6/10) at 2 months prompted two additional sequential sessions. Following the second session, pain decreased to VAS 2/10 with improved healing (Fig. 3D), and after the third session, pain resolved completely (VAS 0/10) coinciding with progressive diploid stability (Fig. 3E). This culminated in a smooth mucosal surface at 3 months (Fig. 3F. i) with diploid DNA-ICM (Fig. 3F. ii), and at 6 months (Fig. 3G), homogeneous pink mucosa with absent keratosis and sustained genomic stability—demonstrating that three ALA-PDT sessions eradicated symptoms and achieved durable remission without recurrence, validating the multi-session protocol to eliminate residual malignant potential despite early biomarker normalization.

## Discussion

PDT is a treatment method based on photochemical reactions, which utilizes visible laser to activate photosensitizing drugs in an oxygenated environment to generate singlet oxygen and radicals. These reactive species can induce cancer cell apoptosis, stimulate immune responses, and cause blood vessel collapse, thereby achieving the purpose of eliminating the lesion. ALA serves as a precursor drug that can be enzymatically converted into protoporphyrin IX (PpIX), a potent photosensitizer. When tongue cancer cells accumulate PpIX and are exposed to 635 nm laser light, PpIX absorbs the laser energy and transitions from the ground state to the excited state. During the transition back to the ground state, excited PpIX transfers energy to surrounding oxygen molecules, converting them into reactive oxygen species [8, 9]. Due to the small dosage of ALA used and its rapid metabolic excretion, patients do not require an extended period of photosensitivity precautions after ALA-PDT. Compared with traditional surgical methods, PDT is a minimally invasive approach that aims to selectively target tumors while sparing normal tissues. Additionally, PDT treatment can be repeated and combined with other therapeutic modalities, with minimal cumulative toxicity to tissues.

ALA-PDT exhibits superior safety and precision over low-dose Foscan [10] and oral ALA-PDT [11] in treating superficial OSCC. By enabling localized PpIX accumulation via tumor-specific metabolism, topical ALA-PDT minimizes systemic toxicity and achieves transient photosensitivity (1–2 days), contrasting with Foscan’s prolonged light-avoidance requirements (1–2 weeks). While Foscan’s 652 nm wavelength allows deeper penetration (≤ 2 cm), its selectivity for superficial OSCC is limited, risking collateral tissue damage. Oral ALA-PDT, though noninvasive, suffers from variable absorption and first-pass metabolism, reducing efficacy. Topical delivery circumvents these limitations, ensuring consistent drug exposure and preserving oral function through minimally invasive treatment. These advantages position topical ALA-PDT as the optimal approach for early-stage OSCC, reserving Foscan for deeper infiltrative lesions.

Nevertheless, the therapeutic efficacy of PDT alone is suboptimal for hyperplastic and highly keratinized lesions due to the limited penetration depth of photosensitizers such as ALA within the affected tissues [12]. Recently, to address this issue, adjuvant treatment modalities involving surface modification using plum-blossom needles [13], CO_2_ lasers (wavelength of 10,600 nm) [14], and diode lasers (wavelength of 980 nm) [15] have been employed. The aim of these methods is to establish epithelial pathways and enhance epithelial absorption of ALA, with clinically significant results observed for these combined approaches. However, each of these strategies possesses inherent limitations. For instance, although effective, manual puncturing with plum-blossom needles lacks precise depth control and can readily induce bleeding, which may metabolize ALA and subsequently diminish therapeutic efficacy. Conversely, CO_2_ lasers and diode lasers exert ablative effects through vaporization, potentially causing submucosal thermal damage and impeding the diffusion of ALA into surrounding tissues [16]. Compared to CO₂ lasers, water-assisted lasers demonstrate superior advantages in minimizing tumor depth penetration during epithelial ablation. By integrating hydrokinetic cooling with precise laser energy delivery, this modality significantly reduces thermal diffusion to adjacent tissues, thereby confining therapeutic effects to targeted hyperplastic regions. Notably, Waterlase operates at a wavelength of 2780 nm, exhibiting extremely high-water absorbance with an action depth of merely 18 micrometers, ensuring remarkable safety. This precise tissue removal eliminates hyperplastic and keratinized epithelial barriers that typically impede photosensitizer and light penetration in conventional PDT. Depth control was achieved through non-contact, continuous fiber tip movement and layer-by-layer ablation, leveraging the high-water absorption at 2780 nm to confine effects to the superficial epithelium while avoiding significant bleeding. The mechanism of Waterlase ablation is based on the high absorption of its 2780 nm wavelength by tissue water, leading to rapid vaporization and micro-explosions that ablate tissue through kinetic energy release rather than thermal conduction. This process minimizes residual heat accumulation, thereby avoiding carbonization and reducing thermal damage to adjacent tissues.

In previous clinical observations, it was noticed that ALA-PDT was unable to completely remove thickened epithelium like leukoplakia. The probable cause lies in the fact that some cells did not display abnormal hyperplasia and were, therefore, insensitive to photodynamic therapy. Taking this into account, we directly employed Waterlase for epithelial surface treatment. This enabled us to eliminate all hyperplastic and thickened epithelial tissues, improve the penetration depth of laser treatment, and consequently augment the therapeutic efficacy of ALA-PDT. Therefore, Waterlase ablation may serve as a useful adjunct to PDT for such lesions. Following epithelial surface ablation, ALA was administered via submucosal injection combined with local wet compress to ensure comprehensive drug coverage of both superficial and deep tissue layers. This approach is designed to bypass the limitations imposed by the epithelial barrier to ALA permeation. ALA-PDT offers minimally invasive tongue cancer treatment by selectively targeting tumors via PpIX activation, sparing normal tissue. Compared to surgery, it reduces functional impairment (e.g., speech/swallowing) and complications (e.g., scarring, infection). Repeatability of PDT allows for combination therapy, achieving tumor remission in early cases. While surgical resection remains critical for advanced tumors, ALA-PDT prioritizes organ preservation and quality of life for superficial lesions.

In Case 1, transient diploid normalization was observed by DNA-ICM following initial ALA-PDT therapy, yet clinical recurrence with renewed aneuploidy emerged at 3 months. This observation suggests that early biomarker improvement may not preclude persistent malignant potential, indicating that single-session treatment fails to eradicate residual aneuploid clones. Within this limited cohort, the achievement of sustained genomic stability and durable remission was associated with the completion of ≥ 3 sequential ALA-PDT sessions (Figs. 1G-H). Thus, multiple sessions are suggested not due to early biomarker-detected failure, but to mitigate the risk of recurrence from residual malignant clones. This prophylactic repetition counteracts the biomarker’s limitation in detecting latent genomic instability, enabling thorough malignant cell eradication to reduce recurrence risk and consolidate therapeutic efficacy. Consistent with this protocol, Cases 2–3 receiving triple-session PDT showed no recurrence at 6-month OSCC follow-up.

Furthermore, DNA-ICM, an advanced cytogenetic method, precisely identifies cells with abnormal chromosome numbers, often associated with tumorigenic states. In soft tissue tumors, particularly OSCC, DNA-ICM exhibits unique diagnostic value, aiding in the differentiation between benign and malignant lesions. DNA quantitation typically uses “c” as the unit, where G0/G1 phase cells are 2c (diploid), and G2/M phase cells are 4c (tetraploid). Clinically, cells with a DNA index ≥ 2.5 (i.e., 5c) are considered the threshold for abnormality. DNA reports focus on DNA aneuploid (index), aneuploid peaks, and cellular proliferation, enhancing diagnostic accuracy and playing a crucial role in early screening, diagnosis, and prognosis of tumors. Post-ALA-PDT, biopsy is discouraged due to potential trauma and complications, making DNA-ICM an ideal alternative for recurrence assessment. Additionally, in thick lesions requiring multiple PDT sessions, DNA-ICM provides a scientific basis for determining treatment frequency [17]. Prior to the treatment, numerous abnormalities were detected in the patient’s DNA-ICM. Upon undergoing one session of ALA-PDT, the DNA-ICM fully recovered to normal. Moreover, no recurrence was witnessed during the half-year follow-up. Based on the clinical recurrence patterns observed in Case 1 and sustained genomic stabilization after triple-session ALA-PDT, we propose the following optimized surveillance protocol for OSCC patient’s post-PDT: DNA-ICM monitoring should be performed monthly during the first year and quarterly from the second year onward. Any detected dysplasia or aneuploidy warrants prompt ALA-PDT reintervention.

In this preliminary case series, the combined use of ALA-PDT, Waterlase ablation, and DNA-ICM showed potential in the management of early-stage OSCC. The synergistic use of these offers a precise, safe, and minimally invasive treatment option for oral tissues. The mechanistic rationale, along with the favorable functional outcomes observed in these cases, warrants further investigation as a potential organ-preserving strategy. Future research will explore optimized combinations and clinical applications of these therapies to achieve optimal patient outcomes and prognosis.

## Data Availability

All data generated or analysed during this study are included in this published article.

## Abbreviations

ALA: 5-aminolevulinic acid
OSCC: Oral squamous cell carcinoma
ALA-PDT: 5-aminolevulinic acid photodynamic therapy
PpIX: Protoporphyrin IX
AI: Artificial intelligence
DNA-ICM: Deoxyribo nucleic acid aneuploidy cytology by image cytometry
EC: Epithelial cells
Lym: Lymphocytes
TCC: Total cell counts
